# Structure-activity relationships and molecular mechanisms of natural products in sepsis-associated organ dysfunction: a narrative review

**DOI:** 10.3389/fphar.2026.1808772

**Published:** 2026-04-10

**Authors:** Jia Wang, Xin Cai, Qun Liang

**Affiliations:** 1 Heilongjiang University of Chinese Medicine, Harbin, China; 2 The First Hospital Affiliated to Heilongjiang University of Chinese Medicine, Harbin, China

**Keywords:** immunometabolism, molecular mechanisms, natural products, network pharmacology, sepsis-associated organ dysfunction

## Abstract

**Background:**

Sepsis is defined as life-threatening organ dysfunction caused by a dysregulated host response to infection. Despite significant advancements in modern life-support technologies, the mortality rate of sepsis and its associated complications in the intensive care unit (ICU) remains unacceptably high.

**Problem:**

The repeated clinical failure of traditional single-target therapies (e.g., anti-endotoxin agents) underscores an urgent need for systemic therapeutic strategies capable of simultaneously restoring the immune, metabolic, and coagulation networks.

**Methods:**

This comprehensive narrative review evaluates the pharmacological mechanisms of natural bioactive compounds in mitigating sepsis-associated organ dysfunction. Unlike previous descriptive reviews, we categorize active compounds based on their core chemical scaffolds (flavonoids, terpenoids, alkaloids, and quinones) and provide a rigorous comparative analysis of their structure-activity relationships (SAR).

**Results:**

Evidence reveals that specific structural features—such as functional group substitutions, spatial ring conformations, and quaternary ammonium charge states—directly dictate the pharmacokinetic stability, blood-brain barrier (BBB) penetrability, and intracellular target affinity of these natural products. By leveraging these structural advantages, natural products exert multi-organ protective effects (spanning the lungs, kidneys, heart, liver, intestines, and brain) through the synergistic modulation of three converging hubs: the NF-κB/NLRP3 inflammatory axis, the Nrf2/HO-1 antioxidant system, and the AMPK/mTOR immunometabolic circuit. Specifically, they demonstrate profound efficacy in inhibiting macrophage M1 polarization, blocking neutrophil extracellular trap (NET) formation, ameliorating mitochondrial bioenergetic crises, and alleviating sepsis-associated encephalopathy (SAE).

**Conclusion:**

Although natural products offer profound multi-target advantages, their clinical translation is frequently hindered by poor druggability, unstable pharmacokinetics, and narrow therapeutic windows. Future research must decisively transition from empirical screening to SAR-guided lead optimization, integrating advanced nano-delivery systems and biomarker-driven precision clinical trials to successfully advance these natural scaffolds into clinical applications.

## 1 Introduction

Sepsis is defined as life-threatening organ dysfunction caused by a dysregulated host response to infection (Singer et al., 2016). This is not merely a clinical definition but represents a global public health crisis. According to data from the Global Burden of Disease study published in *The Lancet*, there were approximately 48.9 million new cases of sepsis worldwide in 2017, leading to about 11 million related deaths, accounting for nearly 20% of global mortality (Rudd et al., 2020). Despite significant advancements in modern critical care technologies, including mechanical ventilation, blood purification, and the use of broad-spectrum antibiotics, the mortality rate for sepsis in intensive care units (ICUs) in developed countries in Europe and America remains high at 30%–38% (Vincent et al., 2019). This high mortality is primarily attributed to the occurrence of SRCs, a cascade of pathological processes involving multiple systems, including sepsis-induced acute lung injury (SILI) (Bos and Ware, 2022), sepsis-associated acute kidney injury (SA-AKI) (Zarbock et al., 2023), sepsis-induced myocardial injury (SIMI) (Carbone et al., 2022), sepsis-associated acute hepatic injury (SAHI) (Beyer et al., 2022), sepsis-associated encephalopathy (SAE), as well as coagulation and gastrointestinal dysfunction. Epidemiological evidence indicates a significant positive correlation between the accumulation of organ dysfunction and patient mortality: SA-AKI is an independent risk factor for death, accounting for 45%–70% of critical care AKI cases (Rudd et al., 2020; Zarbock et al., 2023); furthermore, approximately 40% of sepsis patients develop myocardial depression, and once heart failure occurs, mortality can rise to over 70% (Winterbottom, 2022; Ehrman et al., 2018).

**FIGURE 1:**
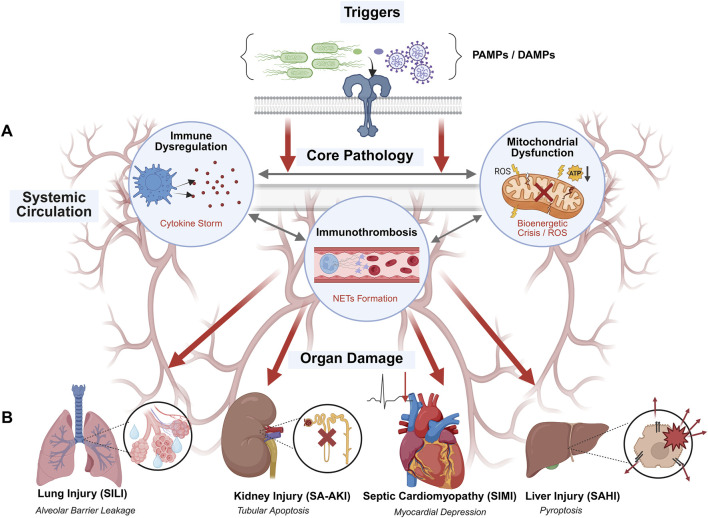
The Systemic Pathological Network of Sepsis-Associated Organ Dysfunction: Interplay of Immune Dysregulation, Metabolic Collapse, and Coagulation Disorders. (Created with BioRender.com.). Note: Sepsis is initiated by the recognition of pathogen-associated molecular patterns (PAMPs, e.g., bacteria, viruses) by innate immune cells (macrophages, neutrophils), triggering a systemic inflammatory cascade. **(A)** Central Pathogenesis: The core pathology involves a triad of disruptions: (1) Immune Dysregulation, characterized by the simultaneous release of pro-inflammatory cytokines (cytokine storm) and immune paralysis; (2) Mitochondrial Dysfunction, leading to cellular bioenergetic crisis and oxidative stress; and (3) Coagulation Abnormalities, driven by endothelial injury and immunothrombosis. **(B)** Organ-Specific Injury: These systemic insults propagate through the circulation, leading to specific organ dysfunction, including ALI/ARDS, SA-AKI, SIMI, and SAHI. The figure illustrates the transition from local infection to systemic multi-organ failure, highlighting the critical role of the “Immune-Metabolic-Coagulation” network.

Why is it still clinically challenging to effectively halt the progression of SRC despite the increasing availability of anti-infective therapies? The fundamental reason lies in the highly complex and systemic nature of the pathophysiological mechanisms underlying sepsis, which cannot be explained by mere pathogen invasion alone. Modern pathophysiological research reveals that the essence of SRC is the comprehensive collapse of the host’s “immune-metabolic-coagulation” network. Pathogen-associated molecular patterns (PAMPs) and damage-associated molecular patterns (DAMPs) excessively activate pattern recognition receptors, triggering a cascade reaction via the NF-κB signaling pathway, which leads to a cytokine storm. This dysregulated inflammatory response not only causes vascular endothelial injury but is also accompanied by a bioenergetic crisis resulting from mitochondrial dysfunction and abnormal activation of the coagulation system (e.g., DIC), ultimately leading to the sequential failure of distant organs such as the lungs, kidneys, heart, and liver, and brain (Sygitowicz and Sitkiewicz, 2020) (Figure 1).

Over the past three decades, drug development based on the concept of “single-target blockade”—such as anti-endotoxin antibodies, specific TNF-α antagonists, or single anticoagulant agents—has repeatedly failed in clinical trials (Marshall, 2014). This phenomenon carries profound cautionary significance: given that sepsis is a systemic disease involving alterations in the expression of thousands of genes at the genomic level, attempting to reverse the condition by blocking a single molecular pathway is unrealistic. This indicates an urgent clinical need for a systemic therapeutic strategy capable of simultaneously modulating the balance of multiple signaling networks and restoring organismal homeostasis, rather than a localized “blocking agent.”

In the search for multi-target modulators, natural products—particularly bioactive small molecules derived from traditional medicine—have re-emerged as a research focus in critical care medicine due to their unique evolutionary advantages and chemical properties (Atanasov et al., 2021). Unlike molecules commonly found in modern synthetic drug libraries, natural products often possess evolutionarily privileged scaffolds that enable them to interact with multiple protein targets in a “polypharmacological” manner, thereby producing synergistic effects (Newman and Cragg, 2020). For example, certain flavonoids have been demonstrated not only to inhibit the transcription of inflammatory factors but also to enhance cellular antioxidant defenses by modulating redox-sensitive proteins and improve mitochondrial metabolic function. In Chinese clinical practice, combination therapies represented by Xuebijing (which contains natural product monomers such as safflower yellow and paeoniflorin) have been supported by substantial clinical evidence showing a significant reduction in mortality among sepsis patients, providing strong real-world evidence for the clinical value of natural products (Zhao et al., 2017).

Beyond sepsis, the usefulness of natural products in clinical practice has also been increasingly recognized in other life-threatening conditions characterized by hyperinflammation, such as the coronavirus disease 2019 (COVID-19) pandemic. For instance, a recent randomized controlled trial demonstrated that adjuvant therapy with xanthohumol in critically ill COVID-19 patients with acute respiratory failure significantly reduced the 28-day mortality rate (from 48% to 20%) and effectively suppressed crucial inflammatory and coagulopathy markers, including IL-6 and D-dimer (Dabrowski et al., 2023). Similarly, to overcome the inherent poor oral absorption of polyphenols, clinical studies utilized a highly bioavailable phospholipid delivery system of quercetin (Quercetin Phytosome®). In COVID-19 outpatients, this optimized formulation accelerated viral clearance, blunted acute-phase reactants (e.g., CRP, Ferritin, and LDH), and significantly lowered the rates of hospitalization and intensive care unit (ICU) admission (Di Pierro et al., 2021a; Di Pierro et al., 2021b). These successful clinical translations in managing virus-induced “cytokine storms” and coagulopathy further underscore the immense potential of optimized natural products in modern critical care medicine.

To ensure the scientific rigor of this review, it is essential to clearly distinguish between the concepts of “natural medicines” and “natural products.” Although traditional medicine often employs crude extracts or compound formulations, the complexity of their constituents and batch-to-batch variability frequently obscure well-defined pharmacological mechanisms. Given that clear structure-activity relationships and pharmacokinetic profiles are crucial for modern drug development, this review will strictly focus on “natural product monomers” with well-defined chemical structures and high purity, rather than on mixtures of undefined composition. This delineation facilitates a more precise evaluation of their drug-like potential and molecular mechanisms.

Although reviews on the treatment of sepsis with natural medicines have been published, most existing literature suffers from simplistic classification logic (merely listing by organ), superficial exploration of mechanisms, and often lacks objective assessment of translational barriers. Unlike previous studies, this article aims to provide a comprehensive narrative review, primarily reflected in the following three aspects: (1) From a chemical structure-based classification perspective, we innovatively introduce a “chemical structure–pharmacological activity” classification system to deeply analyze the fundamental differences in pharmacokinetic behaviors and target selection among flavonoids, terpenoids, alkaloids, and quinones. (2) Construction of a cross-organ core mechanism network, transcending the limitations of single-organ focus, to uncover “shared core mechanisms” spanning the lungs, kidneys, heart, liver, and brain (e.g., the NF-κB/NLRP3 inflammatory axis, the Nrf2/HO-1 antioxidant system, the AMPK/mTOR immunometabolic axis), thereby untangling the molecular principles by which natural products regulate systemic homeostasis. (3) Critical evaluation from a translational medicine perspective, where we not only summarize efficacy but also objectively assess “drug-likeness” bottlenecks, including low bioavailability, narrow therapeutic windows, and heterogeneity in clinical evidence. Through this panoramic analysis, we aim to provide a scientific basis for the future development of novel natural product-based therapies for sepsis and to offer a theoretical framework for overcoming translational barriers from the laboratory to the clinic.

### 1.1 Literature search strategy

To ensure a comprehensive evaluation of the current landscape, an extensive literature search was conducted using databases including PubMed, Web of Science, and Embase, covering publications up to early 2026. The search terms utilized a combination of keywords such as (“sepsis” OR “septic shock” OR “endotoxemia”) AND (“natural products” OR “phytochemicals” OR “flavonoids” OR “alkaloids” OR “terpenoids”) AND (“organ dysfunction” OR “acute kidney injury” OR “cardiomyopathy” OR “acute lung injury” OR “encephalopathy” OR “brain injury”). Articles were primarily selected based on their relevance to molecular mechanisms, structure-activity relationships, and the utilization of well-established *in vivo* and *in vitro* sepsis models.

## 2 Natural products with therapeutic potential in sepsis: a critical analysis of chemical classification and structure-activity relationships

The “multi-target” regulatory effects of natural products within the complex pathological network of sepsis are not random phenomena but are determined by the physicochemical properties of their core chemical scaffolds. A deep understanding of the chemical classification of these compounds not only aids in elucidating their pharmacological mechanisms but also provides a theoretical basis for predicting their pharmacokinetic behaviors (e.g., membrane permeability, metabolic stability) and potential clinical translation barriers. Based on existing literature and chemical structural characteristics, the active compounds currently shown to be effective against SRC are categorized into the following four major classes: polyphenols and flavonoids, terpenoids and saponins, alkaloids, and quinones.

### 2.1 Polyphenols and flavonoids: the interplay between antioxidant defense and bioavailability

Polyphenolic compounds represent the most extensively studied class of natural products in current anti-sepsis research. Their chemical structures share the common feature of containing one or more phenolic hydroxyl groups. These phenolic hydroxyl groups confer an exceptional electron-donating capacity to these compounds, rendering them potent natural antioxidants.

Flavonoids, exemplified by quercetin and naringenin, possess a C2-C3 double bond and an ortho-dihydroxy structure (catechol group) on the B-ring, which constitute the core determinants of their biological activity. This specific chemical architecture not only enables the direct neutralization of reactive oxygen species (ROS) via hydrogen atom transfer but, more importantly, allows these compounds to function as signaling molecules that interact with redox-sensitive proteins within cells (Wang L. et al., 2022). For instance, curcumin, a hydrophobic polyphenol, has been demonstrated to modify cysteine residues on the Keap1 protein through electrophilic reactions, thereby relieving its inhibition of Nrf2 and activating the Nrf2/HO-1 antioxidant pathway (Xu et al., 2019). This mechanism elucidates why polyphenolic compounds exhibit superior efficacy in counteracting sepsis-induced “oxidative burst.” Their action extends beyond mere radical scavenging to the initiation of endogenous enzymatic defense systems within cells. Furthermore, resveratrol, by mimicking caloric restriction effects and activating the SIRT1 deacetylase, demonstrates unique structure-activity advantages in improving mitochondrial function and inhibiting ferroptosis (Zeng et al., 2023).

While polyphenolic compounds exhibit potent anti-inflammatory and antioxidant activities *in vitro*, their clinical application must be critically evaluated from a pharmacokinetic perspective. Polyphenols are generally subject to a “bioavailability paradox”: their abundant phenolic hydroxyl groups, which are the source of their bioactivity, also render them highly susceptible to glucuronidation or sulfation metabolism in the liver and intestine (first-pass effect), resulting in extremely low concentrations of the parent compound entering systemic circulation (Anand et al., 2007). For instance, numerous studies have shown that the plasma concentration of curcumin after oral administration is difficult to achieve the effective concentration observed *in vitro*. This implies that the future research focus should not remain solely on validating their anti-inflammatory phenotypes. Instead, it is crucial to delve into strategies for protecting their phenolic hydroxyl groups from premature metabolism, such as through structural modifications (e.g., methylation to enhance metabolic stability) or nano-delivery systems, which is essential for improving their drug-likeness. Recent clinical evidence strongly supports this formulation-based approach. For example, a newly published randomized controlled trial demonstrated that employing a micellar formulation for xanthohumol, a highly bioactive prenylated chalcone, increased its oral bioavailability in humans by approximately 9-fold compared to its native form (Brehmer-Henkel et al., 2026). Such advanced delivery technologies represent a critical translational pathway to overcoming the pharmacokinetic limitations of polyphenols, ensuring that their potent *in vitro* efficacy can be reliably replicated in clinical settings.

#### 2.1.1 Comparative SAR and pharmacokinetic implications of flavonoids

To truly harness the therapeutic potential of flavonoids in sepsis, a comparative understanding of their SAR and pharmacokinetic stability is essential. While the aglycone quercetin exhibits potent *in vitro* anti-inflammatory and antioxidant activities—primarily driven by the 2,3-double bond of the C-ring and the catechol B-ring (3',4'-dihydroxy groups)—its clinical translation is frequently hindered by rapid first-pass metabolism and poor pharmacokinetic stability (Chang et al., 2013; Loke et al., 2008). Specific functional group modifications profoundly dictate the balance between *in vivo* efficacy and toxicity profiles. For instance, glycosylation at the C3 position (e.g., converting quercetin to rutin) substantially attenuates direct antioxidant capacity and delays absorption, as rutin requires prior hydrolysis by cecal microflora (Zeng et al., 2020; Manach et al., 1997). Similarly, the aglycone hesperetin demonstrates more robust anti-inflammatory activity than its glycoside derivative, hesperidin (Choi et al., 2022).

However, this structural modification presents a critical pharmacological trade-off. While increased lipophilicity (lower degree of glycosylation) enhances cellular uptake and receptor binding affinity, it concurrently increases cellular toxicity at higher concentrations (Choi et al., 2022). Highly water-soluble glycosides exhibit the lowest cytotoxicity, thereby widening the narrow therapeutic window (Choi et al., 2022). Furthermore, specific methylation (e.g., 3'-O-methyl-quercetin) preserves substantial ICAM-1 inhibitory activity while altering the compound’s lipophilicity (LogP), which governs intestinal accessibility and bioavailability along a bell-shaped curve (Lotito et al., 2011; Maeda et al., 2026). Therefore, instead of merely utilizing flavonoids with maximal free hydroxyl groups, strategically applying structural analogs with specific methylation or glycosylation is required to overcome the “bioavailability paradox”, ultimately optimizing systemic distribution and mitigating dose-dependent toxicity during the cytokine storm of sepsis.

### 2.2 Terpenoids and saponins: lipophilicity advantages and precise modulation of intracellular targets

In contrast to polyphenols, terpenoids are synthesized via the polymerization of isoprene units and typically exhibit strong lipophilicity. This physicochemical property enables them to readily penetrate the bilayer lipid structure of the cell membrane, allowing for direct interaction with intracellular signaling proteins or nuclear receptors. This characteristic confers a natural advantage in the treatment of sepsis, a condition involving dysregulation of multiple intracellular signaling pathways.

Tanshinone IIA (Tan IIA) is a typical diterpenoid quinone compound. Its rigid lipophilic phenanthrenequinone skeleton enables it to rapidly enter macrophages and specifically target and block the cytoplasmic ROCK2/NF-κB signaling axis (Liu J. et al., 2022). This efficient transmembrane capability explains its significant efficacy in regulating macrophage polarization (shifting from pro-inflammatory M1 to reparative M2 phenotypes), thereby effectively reversing sepsis-induced alveolar epithelial damage. Similarly, the sesquiterpene lactones parthenolide and abietic acid also demonstrate potent inhibitory effects on the intracellular NF-κB pathway, confirming the superiority of lipophilic skeletons in targeting intracellular transcription factors (Zhang et al., 2025).

Triterpenoid saponins, such as ginsenoside Rg1 and astragaloside IV (AS-IV), possess a tetracyclic triterpene scaffold whose spatial conformation closely resembles that of endogenous glucocorticoids in the human body. This structural feature provides a crucial clue for explaining their pharmacological mechanisms: they can exert potent anti-inflammatory effects in a hormone-like manner—for instance, by inhibiting NLRP3 inflammasome assembly and activating SIRT1—yet are often not accompanied by the severe side effects commonly associated with glucocorticoids, such as broad immunosuppression or osteoporosis (Hu et al., 2024; Sun et al., 2023). This “hormone-mimetic” yet selective immunomodulatory property suggests that saponins may be ideal candidate drugs for replacing high-dose hormone therapy in the treatment of late-stage sepsis characterized by immune paralysis or excessive inflammatory responses, warranting focused attention in clinical translation.

Furthermore, the exceptional lipophilicity of terpenoids confers a distinct therapeutic advantage in addressing SAE, delirium, and subsequent long-term dementia—critical and devastating complications in the intensive care unit. The ability of small molecules to penetrate the blood-brain barrier (BBB) is heavily dependent on optimal molecular size and lipophilicity (LogP) (Song et al., 2026; Song et al., 2023). For instance, the lipophilic nature of tanshinone IIA and the sesquiterpene lactone parthenolide allows them to effectively cross the BBB (Xiong et al., 2019; Wang X. et al., 2022). Once in the central nervous system, they act as potent neuroprotectants by inhibiting microglial over-activation, mitigating neuroinflammation via the TLR4/NF-κB and NLRP3 pyroptosis pathways, and simultaneously upregulating tight junction proteins (e.g., Claudin-5, ZO-1) to restore or even enhance BBB integrity (Yue et al., 2021; Jin et al., 2020; Wang Y. et al., 2018). Parthenolide has also been shown to significantly ameliorate cognitive dysfunction and memory deficits in dementia and Alzheimer’s disease models, addressing a major long-term societal burden for sepsis survivors (Hu et al., 2025). Interestingly, while triterpenoid saponins (such as ginsenosides) present a pharmacological paradox due to their larger molecular size and higher polarity, they still achieve substantial brain penetration. Rather than relying solely on passive diffusion, they utilize active transporter-mediated uptake (such as GLUT1) or undergo gut microbiota-mediated deglycosylation into highly lipophilic active metabolites (e.g., Compound K) (Ren et al., 2023; Kong et al., 2024). Consequently, these natural products not only suppress acute neuroinflammation and delirium but also protect against chronic amyloid and tau-related neurotoxicity, positioning them as highly promising multifunctional agents for critical neurological care (Jiang et al., 2025; Wu et al., 2022).

#### 2.2.1 Comparative SAR and spatial conformational analysis of diterpene quinones

Beyond simple functional group substitutions, the spatial conformation of natural products fundamentally dictates their target binding affinities and *in vivo* specificities. A compelling paradigm is the comparison between tanshinone IIA and cryptotanshinone. The primary structural distinction lies in the D-ring saturation state: tanshinone IIA possesses an aromatic furan ring with a C15-C16 double bond, whereas cryptotanshinone features a saturated dihydrofuran ring (Roth et al., 2023; Song et al., 2022). This seemingly minor dehydrogenation significantly alters the 3D molecular geometry. The double bond in tanshinone IIA enforces a highly rigid, planar phenanthrene-furan fully conjugated system, which optimally fits into specific planar hydrophobic pockets or DNA minor grooves (Zhang et al., 2008; Wu et al., 1991). Conversely, the saturation in cryptotanshinone disrupts this resonance, creating a slightly puckered, non-planar conformation (Zhang et al., 2008).

Crucially, molecular docking and cell-free assays reveal that this non-planar spatial distortion endows cryptotanshinone with functionally superior binding affinities for specific pro-inflammatory enzymes. For instance, cryptotanshinone demonstrates potent inhibition against mPGES-1 (IC_50_ = 1.9 μM) and 5-lipoxygenase (IC_50_ = 7.1 μM), whereas the planar tanshinone IIA exhibits negligible inhibition against these specific targets (Saviano et al., 2022). Furthermore, these structural nuances govern their pharmacokinetic interconversion. The saturated dihydrofuran ring renders cryptotanshinone chemically more susceptible to spontaneous oxidation, rapidly converting to tanshinone IIA under physiological conditions (Roth et al., 2023). This indicates that cryptotanshinone functions, in part, as a prodrug *in vivo* (Song et al., 2007). Despite this instability, animal models consistently show that cryptotanshinone produces a more pronounced dose-dependent suppression of the NF-κB p65 complex and superior systemic anti-inflammatory outcomes compared to tanshinone IIA (Maione et al., 2018). Thus, modulating the planarity of the furano-o-quinone scaffold not only shifts the pharmacological target specificity but also determines the metabolic trajectory of these diterpenoids during the hyperinflammatory phase of sepsis.

### 2.3 Alkaloids: mitochondrial targeting and metabolic reprogramming

Alkaloids are a class of nitrogen-containing organic compounds in which the nitrogen atom typically exhibits basicity and is often protonated to carry a positive charge under physiological pH conditions. This chemical characteristic holds particular strategic significance for the treatment of sepsis, especially in interventions targeting mitochondrial dysfunction.

Due to the highly negative potential (membrane potential ΔΨm) inside mitochondria relative to the cytoplasm, lipophilic cations carrying a positive charge tend to accumulate within the mitochondrial matrix. Taking the isoquinoline alkaloid berberine as an example, studies have confirmed its ability to significantly improve mitochondrial structure and function in septic cardiomyopathy and increase ATP synthesis (Shen et al., 2024). This is not a coincidental finding but rather a “mitochondrial targeting” effect determined by its chemical properties. This accumulation enables it to directly modulate the activity of mitochondria-associated metabolic enzymes, thereby protecting high-metabolism organs (such as the heart) from energy crises.

More critically, alkaloids exhibit unique advantages in regulating immunometabolism. Both phellodendrine and berberine have been identified as potent activators of the AMPK pathway (Ding et al., 2025). As a key sensor of cellular energy homeostasis, activation of AMPK not only inhibits anabolic pathways (such as mTOR) but also forces macrophages to shift from the hyper-glycolytic state (Warburg effect) characteristic of the acute phase of sepsis back to oxidative phosphorylation (OXPHOS) via metabolic reprogramming (O'Neill et al., 2016). In sepsis, a disease fundamentally characterized by “immune-metabolic dysregulation,” this “metabolic correction” capability of alkaloids targets the energetic foundation of inflammation. Its therapeutic value may therefore surpass that of anti-inflammatory agents that merely block downstream inflammatory cytokines.

Beyond intracellular metabolic regulation, alkaloids also exert critical endothelial-protective effects. Increased vascular permeability is a fundamental pathology driving fluid resuscitation refractoriness and organ edema in patients with sepsis and septic shock. Recent clinical evidence demonstrates that berberine can significantly modulate Vascular Endothelial Growth Factor (VEGF) concentrations and improve vascular function parameters in patients with metabolic dysfunction (Koperska et al., 2025). By targeting the endothelial barrier and regulating VEGF, berberine holds the immense potential to correct sepsis-induced microvascular hyperpermeability, perfectly synergizing with its intracellular mitochondrial protection.

#### 2.3.1 Comparative SAR and mitochondrial targeting of alkaloids

The protoberberine alkaloids provide a fascinating paradigm of how the nitrogen oxidation state and specific ring substitutions dictate subcellular localization, pharmacokinetic profiles, and toxicity in sepsis-induced organ injury. The fundamental structural divergence between berberine and phellodendrine lies in their amine structures: berberine is a quaternary ammonium alkaloid with a permanently charged nitrogen, whereas phellodendrine is a tertiary amine (Luo et al., 2024; Jiang et al., 2026; Tian et al., 2020; Chen et al., 2010). This permanent cationic nature at physiological pH is the primary driver for berberine’s exceptional mitochondrial tropism. Driven by the highly negative mitochondrial membrane potential, the quaternary ammonium structure enables berberine to selectively traverse lipid bilayers and accumulate within the mitochondrial matrix at concentrations far exceeding cytosolic levels (Fang X. et al., 2022; Pereira et al., 2007; Pereira et al., 2008). Once localized, it exerts potent organelle-specific protective effects, such as directly binding to the juxtamembrane loop domain of the mitochondrial calcium uniporter (MCU) to prevent rapid Ca^2+^ overload, and modulating mitochondrial complex I via SIRT3-dependent mechanisms (Zhao et al., 2025; Wang et al., 2025).

In contrast, tertiary amines like phellodendrine exist in a pH-dependent equilibrium. While their deprotonated forms can readily cross cellular membranes, they lack the charge-driven, hyper-selective mitochondrial accumulation observed with berberine, leading to distinct hepatic disposition and systemic distribution profiles (Tian et al., 2020; Burckhardt and Thelen, 1995; Inácio et al., 2013). Furthermore, subtle functional group modifications on the protoberberine scaffold dramatically shift the margin between therapeutic efficacy and toxicity. For instance, the 2,3-methylenedioxy bridge on the A-ring of berberine is critical for optimal organ protection. The demethylenated metabolite, berberrubine, paradoxically induces severe cPLA2-mediated nephrotoxicity, an effect that the parent berberine specifically inhibits (Rao et al., 2024). This highlights that the quaternary ammonium moiety and intact methylenedioxy structures are not mere structural bystanders, but critical determinants that guide selective mitochondrial binding affinity while restricting accumulation-related toxicity during septic bioenergetic crises.

### 2.4 Quinones and others: the balance between redox cycling and toxicity

The chemical core of quinone compounds is the conjugated diketone structure within their molecules. This unique electronic structure endows them with the ability to undergo reversible “redox cycling” within cells, making them a double-edged sword for regulating cellular redox status (Bolton et al., 2000).

From a pharmacological mechanism perspective, quinone compounds act as electrophiles and can undergo Michael addition reactions with the cysteine thiol groups of intracellular proteins. For example, emodin, a typical anthraquinone compound, has been demonstrated to significantly alleviate sepsis-induced lung and kidney damage by downregulating TNF-α/IL-6 and improving blood gas parameters (Shang et al., 2021). This mechanism may involve the activation of defense pathways such as Nrf2 through electrophilic modification, thereby inducing the production of antioxidant enzymes in cells (i.e., “hormesis”). Furthermore, piceatannol (a stilbene compound structurally similar to a quinone precursor) utilizes its structural properties to specifically target and inhibit the JAK2/STAT3 pathway, thereby blocking pyroptosis in sepsis-induced cardiomyopathy (Xie et al., 2021). Crucially, the therapeutic spectrum of piceatannol extends to systemic metabolic complications. Stress-induced hyperglycemia and insulin resistance are common and severe challenges in the clinical management of septic patients. Clinical trials have confirmed that piceatannol supplementation can effectively correct metabolic health and improve insulin sensitivity (Kitada et al., 2017). For clinicians, the ability of such compounds to simultaneously curb inflammatory pyroptosis in vital organs and correct systemic septic insulin resistance highlights their immense dual-target clinical utility. Meanwhile, cryptotanshinone has been shown to inhibit key enzymes of glycolysis in macrophages, cutting off the inflammatory supply at its metabolic source (Ye et al., 2023).

However, if the redox cycle becomes dysregulated, quinones can generate excessive ROS and deplete intracellular reduced glutathione (GSH), leading to severe oxidative damage and even hepatorenal toxicity. Therefore, in the development of such drugs, defining their “therapeutic window” is more critical than merely pursuing high activity. Future studies must establish a rigorous dose–response relationship to ensure that while activating protective pathways, these agents do not induce subsequent oxidative damage, particularly in the pathological context of sepsis patients who already present with organ dysfunction.

In summary, the diverse chemical scaffolds of natural products provide a material basis for their multi-target pharmacological activities in the treatment of sepsis. Polyphenols exert antioxidant effects through phenolic hydroxyl groups but are limited by bioavailability; terpenoids utilize their lipophilic properties to penetrate cell membranes and target intracellular signaling; alkaloids leverage their positive charge to target mitochondria and remodel immunometabolism; while quinones exert bidirectional effects through redox modulation. This intrinsic “structure-function” relationship not only explains the differing emphases of various compound types on antioxidant, immunomodulatory, or metabolic reprogramming effects, but also provides a theoretical basis for the clinical design of “combination therapies” based on complementary mechanisms. To more systematically present the preclinical evidence levels and molecular mechanisms of representative compounds, we have summarized the relevant research details—including chemical classification, study models, and molecular targets—in the following table (see Table 1).

**TABLE 1:** Summary of experimental evidence for natural products in sepsis-associated organ dysfunction: Chemical classification, models, and molecular targets.

Class	Compound	Target organ	Key mechanisms and targets	Model	Effective dose/Concentration	Ref.
Polyphenols, Flavonoids and Glycosides	Quercetin	Lung	Targets: Inhibits NF-κBEffects: Decreases inflammatory cell infiltration and oxidative stress (reduces MDA, increases SOD/CAT/GSH-Px)	*In vivo* (LPS Rats)	*In vivo*: 50 mg/kg (p.o.)	Huang et al. (2015)
	Quercetin	Lung	Targets: Induces HO-1 expression; Decreases MMP-9 activityEffects: Suppresses production of TNF-α, IL-1β, and IL-6; Reduces pulmonary edema	*In vivo* (LPS Mice); *In vitro* (AMJ2-C11, LA-4 cells)	*In vivo*: 10 μM (50 μL, i.t.)*In vitro*: 10–20 μM	Takashima et al. (2014)
	Quercetin	Lung	Targets: Downregulates ALOX5; Activates PI3K/AKT pathwayEffects: Represses LPS-induced cardiomyocyte apoptosis, inflammation, and ferroptosis (regulates Fe2+, ROS, GSH, GPX4)	*In vitro* (LPS-induced AC16 human cardiomyocytes)	*In vitro*: 15–45 μM	Guan et al. (2024)
	Puerarin	Lung	Targets: Activates LXRα; Inhibits NF-κB activationEffects: Reduces MPO activity; Suppresses production of TNF-α, IL-1β, and IL-6; Attenuates lung edema	*In vivo* (LPS Mice); *In vitro* (LPS-induced RAW264.7, A549 cells)	*In vivo*: 25–100 mg/kg (i.p.)*In vitro*: 10–40 μM	Wang X. et al. (2018)
	Curcumin	Lung	Targets: Inhibits NF-κB pathwayEffects: Reduces MDA, TNF-α, IL-6; Increases SOD; Attenuates lung lesion and apoptosis	*In vivo* (IIR Rats)	*In vivo*: 1–5 mg/kg (p.o.)	Fan et al. (2015)
	Resveratrol	Heart	Targets: Activates SIRT1/Nrf2 pathwayEffects: Anti-ferroptosis (regulates ACSL4, GPX4, SLC7A11); Reduces ROS and MDA; Improves mitochondrial function	*In vivo* (CLP Rats); *In vitro* (LPS-induced H9c2 cells)	*In vivo*: 30 mg/kg (i.p.)*In vitro*: 10–40 μM	Zeng et al. (2023)
	Salvianolic acid B	Heart	Targets: Enhances ATF5-mediated UPRmtEffects: Improves mitochondrial dysfunction; Reduces apoptosis, ROS, and mitochondrial fission	*In vivo* (LPS Mice); *In vitro* (LPS-induced cardiomyocytes)	*In vivo*: 10–60 mg/kg (i.p.)*In vitro*: 10 μM	Chen et al. (2024)
	Naringenin	Heart	Targets: Targets HIF-1αEffects: Reduces cardiomyocyte apoptosis, necroptosis (RIP3, p-MLKL), and pro-inflammatory cytokines (IL-1β, MCP-1, TNF-α)	*In vivo* (LPS Mice); *In vitro* (LPS-stimulated HL-1/H9c2 cells)	*In vivo*: 100 mg/kg (i.p.)*In vitro*: 2.5–10 μM	Pan et al. (2024)
	Hesperetin	Intestine	Targets: Inhibits ROS/autophagy pathwayEffects: Reduces NETs formation and PMN infiltration; Protects tight junctions (claudin-1, occludin); Switches PMN death to apoptosis	*In vivo* (LPS Mice); *In vitro* (PMA-induced PMNs)	*In vivo*: 50 mg/kg (i.p.)*In vitro*: 10–100 μM	Chen et al. (2023)
	Fortunellin	Kidney	Targets: Inhibits TLR4/NF-κB pathwayEffects: Anti-ferroptosis (reduces MDA, Fe2+; increases SOD, GSH); Reduces p-p65, p-IκBα, p-IRAK4	*In vivo* (CLP Mice); *In vitro* (LPS-induced HK-2 cells)	*In vivo*: 30 mg/kg (p.o.)*In vitro*: 20–80 μM	Tianzhi et al. (2025)
	Piceatannol	Heart	Targets: Inhibits JAK2/STAT3 pathwayEffects: Reduces apoptosis (decreases Bax, Cleaved-Caspase-3); Reduces inflammation (TNF-α, IL-6, IL-1β) and oxidative stress	*In vivo* (CLP Mice); *In vitro* (LPS-induced H9C2 cells)	*In vivo*: 20 mg/kg (p.o.)*In vitro*: 10–40 μM	Xie et al. (2021)
	6-Gingerol	Liver	Targets: Activates Nrf2/HO-1 pathwayEffects: Inhibits pyroptosis (reduces NLRP3, Caspase-1, IL-1β); Reduces AST, ALT, and ROS.	*In vivo* (CLP Mice); *In vitro* (LPS + ATP-induced RAW 264.7 cells)	*In vivo*: 40 mg/kg (p.o.)*In vitro*: 8 μM	Hong et al. (2020)
	Paeonol	Liver	Targets: Inhibits NF-κB nuclear translocationEffects: Improves mitochondrial function; Reduces ROS, MDA, ALT, AST; Decreases apoptosis	*In vitro* (LPS-induced L02 human hepatocytes)	*In vivo*: N/AIn vitro: 1–10 μg/mL	Xu et al. (2021)
	Forsythiaside B	Coagulation/Multi-organ	Targets: Downregulates PAD4 expressionEffects: Inhibits NETs formation; Ameliorates coagulopathy (increases PLT, FIB; decreases vWF, TM, TAT); Reduces inflammation	*In vivo* (CLP Rats); *Ex vivo* (PMA-induced neutrophils)	*In vivo*: 20–80 mg/kg (i.v.)*In vitro*: N/A	He et al. (2022)
	OPC	Kidney	Targets: Activates PI3K/AKT; Inhibits NF-κB pathwayEffects: Attenuates oxidative stress (increases SOD/CTA/GSH, decreases MDA); Inhibits inflammation (decreases TNF-α, IL-1β, IL-6) and apoptosis	*In vivo* (LPS Mice); *In vitro* (LPS + ATP-induced MTECs)	*In vivo*: 2.5–10 mg/kg (p.o.)*In vitro*: 2.5–5 μM	Cui et al. (2025)
	Salidroside	Liver	Targets: Activates SIRT1; Inhibits NF-κB & NLRP3 inflammasomeEffects: Protects liver function (reduces AST, ALT); Decreases pyroptosis (GSDMD, Cleaved Caspase-1) and oxidative stress	*In vivo* (LPS Mice)	*In vivo*: 50 mg/kg (i.p.)*In vitro*: N/A	Meng et al. (2024)
Terpenoids and Saponins	Eriocitrin	Lung	Targets: Upregulates MKP1; Inactivates MAPK (ERK/JNK/p38)Effects: Inhibits glycolysis (downregulates HIF-1α, PFKFB3, HK2, PKM2); Reduces ROS, MDA, and pro-inflammatory cytokines	*In vivo* (LPS Mice); *In vitro* (LPS-induced peritoneal macrophages)	*In vivo*: 10–60 mg/kg (p.o.)*In vitro*: 2–10 μM	Li et al. (2023)
	Tanshinone IIA	Lung	Targets: Downregulates ROCK2; Inactivates NF-κB pathwayEffects: Reduces apoptosis (decreases Bax/Cleaved-caspase-3); Reduces neutrophil infiltration (MPO) and lung edema	*In vivo* (CLP Rats); *In vitro* (LPS-induced RLE-6TN cells)	*In vivo*: 5–20 mg/kg (i.p.)*In vitro*: 10 μM	30 ( Liu J. et al., 2022 )
	Tanshinone IIA	Liver	Targets: Activates SIRT1/Sestrin2/HO-1 pathwayEffects: Protects liver function (reduces AST, ALT); Decreases inflammation (IL-1β, TNF-α) and apoptosis (Bax/Bcl-2 ratio)	*In vivo* (CLP Mice); *In vitro* (LPS-induced AML12, L-02 cells)	*In vivo*: 30 mg/kg (p.o.)*In vitro*: 5 μM	Tian et al. (2025)
	Cryptotanshinone	Lung	Targets: Activates AMPK pathwayEffects: Induces M2 macrophage polarization; Reverses metabolic switch (inhibits glycolysis, promotes FAO); Reduces pulmonary edema	*In vivo* (LPS Rats); *In vitro* (LPS-induced RAW264.7 cells)	*In vivo*: 15–60 mg/kg (p.o.)*In vitro*: 2.5–10 μM	Ye et al. (2023)
	Astragaloside IV	Kidney	Targets: Activates SIRT1; Negatively regulates Gpr97-TPL2 signalingEffects: Protects against S-AKI and HHcy-exacerbated S-AKI; Reduces tubular inflammation, oxidative stress (MDA, ROS), and apoptosis	*In vivo* (CLP Mice; LPS + HHcy Mice); *In vitro* (LPS + Hcy-induced NRK-52E cells)	*In vivo*: 100 mg/kg (i.p.) [Zhang]; p.o. [Xu]*In vitro*: N/A	Xu J et al. (2024); Zhang (2022)
	Astragaloside IV	Liver	Targets: Modulates Nrf2 & NLRP3 pathwaysEffects: Attenuates oxidative stress (increases HO-1, NQO1, SOD); Inhibits inflammation (decreases NLRP3, Caspase-1, IL-1β) and liver injury	*In vivo* (LPS Mice); *In vitro* (LPS-induced AML-12 cells)	*In vivo*: 80 mg/kg (i.p.)*In vitro*: 20–80 μM	Sun et al. (2023)
	Ginsenoside Rg1	Kidney	Targets: Activates SIRT1; Suppresses NF-κB pathwayEffects: Nephroprotective; Reduces oxidative stress (MDA, 4-HNE, PC) and apoptosis (Bax, Cleaved Caspase-3); Decreases pro-inflammatory cytokines	*In vivo* (LPS Mice); *In vitro* (LPS-induced HK-2 cells)	*In vivo*: 200 mg/kg (i.p.)*In vitro*: 10–50 μM	Hu et al. (2024)
	Ginsenoside Rg1	Heart	Targets: Regulates FAK/AKT-FOXO3A pathwayEffects: Inhibits ferroptosis (reduces Fe2+); Reduces apoptosis (promotes Bcl-2) and inflammation (TNF-α, IL-1β); Alleviates SIMD.	*In vivo* (CLP Mice); *In vitro* (LPS-induced H9c2 cells)	*In vivo*: 35–70 mg/kg (i.p.)*In vitro*: 25 μM	Lin et al. (2024)
	Carvacrol	Heart	Targets: Inhibits TLR4/MyD88/NF-κB and MAPK pathways; Inhibits NLRP3Effects: Reduces pyroptosis (decreases GSDMD, Caspase-1); Attenuates oxidative stress and apoptosis	*In vivo* (LPS Mice); *In vitro* (LPS-induced H9c2 cells)	*In vivo*: 25–100 mg/kg (p.o.)*In vitro*: 0.75–3 μM	Xu L et al. (2024)
	Parthenolide	Coagulation/Endothelium	Targets: Upregulates BRD4/BCL-XL pathwayEffects: Inhibits mitochondrial-mediated apoptosis; Reduces mitochondrial fragmentation and ROS; Improves coagulopathy	*In vivo* (CLP Rats); *In vitro* (LPS-induced VECs)	*In vivo*: 5 mg/kg (i.v.)*In vitro*: 1 μM	Zhang et al. (2025)
	Ginkgolide A	Kidney	Targets: Upregulates miR-25; Targets Nox4Effects: Attenuates oxidative stress; Reduces inflammation (TNF-α, IL-1β, IL-6) and apoptosis (Bax, Cleaved Caspase-3/8)	*In vivo* (LPS Mice); *In vitro* (LPS-induced NRK-52E cells)	*In vivo*: 20 mg/kg (i.v.)*In vitro*: 5–40 μg/mL	Li et al. (2021)
	Hederagenin	Lung	Targets: Inhibits NLRP3 and NF-κBEffects: Suppresses M1 macrophage polarization; Reduces inflammatory cell infiltration and TNF-α/IL-6	*In vivo* (CLP Rats); *In vitro* (LPS-induced THP-1 macrophages)	*In vivo*: 12.5–50 mg/kg (p.o.)*In vitro*: 100 μM	Wang and Zhao (2022)
	Taraxasterol	Lung	Targets: Inhibits NF-κBEffects: Reduces M1 macrophage polarization; Reduces MPO and increases SOD/CAT; Alleviates lung injury	*In vivo* (LPS Rats)	*In vivo*: 8 mg/kg (p.o.)*In vitro*: N/A	Bu et al. (2022)
	Abietic acid	Lung	Targets: Downregulates NF-κB pathwayEffects: Inhibits M1 macrophage polarization; Reduces IL-1β, TNF-α, IL-6, MIP-2	*In vivo* (CLP Mice); *In vitro* (LPS-induced RAW264.7 cells)	*In vivo*: 40 mg/kg (i.p.)*In vitro*: 20–80 μM	Fang H et al. (2022)
	Shionone	Kidney	Targets: Inhibits ECM1; Activates GM-CSF/STAT5/Arg1Effects: Promotes M2 macrophage polarization; Alleviates sepsis-induced AKI.	*In vivo* (CLP Mice); *In vitro* (LPS-induced RAW264.7 cells)	*In vivo*: 50–100 mg/kg (p.o.)*In vitro*: 0.5–2.0 μg/mL	Zhang et al. (2022)
	Iso-seco-tanapartholide	Lung	Targets: Inhibits PFKFB3-mediated glycolysisEffects: Inactivates JAK/STAT3 and NF-κB pathways; Decreases ECAR & lactate; Reduces pro-inflammatory cytokines and airway inflammation	*In vivo* (LPS Mice); *In vitro* (LPS-induced RAW264.7 & BMDMs)	*In vivo*: 10–20 mg/kg (i.p.)*In vitro*: 0.5–2 μM	Kong et al. (2023)
Alkaloids	Berberine	Heart	Targets: Upregulates Notch1/NICD; Inhibits TLR4/NF-κBEffects: Preserves mitochondrial ultrastructure & ATP content; Reduces cardiomyocyte apoptosis, swelling and inflammation; Improves cardiac hemodynamics (LVEF, ±dp/dt max) and reduces cTnT	*In vivo* (CLP Rats; LPS Rats); *In vitro* (LPS-induced H9C2 cells)	*In vivo*: 50 mg/kg (p.o.)*In vitro*: 50 μM	Shen et al. (2024), Chen et al. (2021)
	Phellodendrine	Intestine	Targets: Activates AMPK; Inhibits mTOR signalingEffects: Promotes autophagy (increases LC3II, Beclin1; reduces p62); Suppresses oxidative stress (increases SOD2, CAT, GSH); Reduces intestinal apoptosis	*In vivo* (Burn + LPS-induced sepsis Mice)	*In vivo*: 30 mg/kg (i.p.)*In vitro*: N/A	Ding et al. (2025)
	Paclitaxel	Kidney	Targets: Regulates lnc-MALAT1/miR-370–3p/HMGB1 axisEffects: Reduces cell apoptosis and promotes proliferation; Inhibits inflammation (decreases TNF-α, IL-6, IL-1β)	*In vitro* (LPS-induced HK-2 cells); Clinical (Serum from sepsis patients)	*In vivo*: N/AIn vitro: 5 mg/kg (Unit as per original text)	Xu et al. (2020)
Quinones, Polysaccharides and Others	Emodin	Lung	Targets: Suppresses pro-inflammatory & oxidative pathwaysEffects: Reduces pulmonary edema (W/D) and MPO; Downregulates TNF-α, IL-1β, IL-6, IL-18; Improves gas exchange (PaO2​,PaCO2​)	*In vivo* (LPS, CLP, or SAP-induced ALI Mice/Rats)	*In vivo*: 5–100 mg/kg (p.o./i.p.)*In vitro*: N/A	Liu et al. (2024)
	Emodin	Kidney	Targets: Upregulates Nrf2 and AUF1Effects: Enhances anti-oxidative capacity (increases CAT, GSH-PX, T-SOD, decreases MDA); Suppresses inflammation (decreases PCT, IL-6, NGAL) and protects renal function	*In vivo* (CLP Rats)	*In vivo*: 20–60 mg/kg (p.o.)*In vitro*: N/A	Ding X (2023)
	Plumbagin	Heart	Targets: Inhibits JAK2/STAT3 pathwayEffects: Attenuates cardiomyocyte pyroptosis (decreases Caspase-11, GSDMD, GSDMD-N); Reduces oxidative stress (MDA, ROS) and inflammation (IL-1β, IL-18); Improves cardiac function	*In vivo* (CLP Mice)	*In vivo*: 2–4 mg/kg (i.p.)*In vitro*: N/A	Du et al. (2024)
	Prim-O-glucosylcimifugin	Liver	Targets: Inhibits NLRP3 inflammasomeEffects: Reduces macrophage infiltration; Promotes M2 macrophage polarization; Reduces IL-1β, IL-18, and hepatocyte apoptosis	*In vivo* (CLP Mice); *In vitro* (LPS + ATP-induced primary peritoneal macrophages)	*In vivo*: 10–50 mg/kg (i.p.)*In vitro*: 10–100 mg/mL (Unit as per original text)	Liu L et al. (2022)
	Byakangelicin	Kidney	Targets: Binds to PSMC5; Inhibits TLR4/NF-κB pathwayEffects: Reduces inflammation (TNF-α, IL-6, IL-1β, MCP-1) and apoptosis (downregulates BAX, upregulates BCL-2)	*In vivo* (CLP Mice); *In vitro* (LPS-induced HK-2 cells)	*In vivo*: 25–50 mg/kg (i.p.)*In vitro*: 2–8 μM	Hangda et al. (2024)
	PPPS	Heart	Targets: Upregulates p110β and its interaction with PTEN; Inhibits PI3K/AKT/NF-κB pathwayEffects: Reduces myocardial enzymes (AST, LDH, CK-MB); Attenuates oxidative stress, apoptosis, and inflammation	*In vivo* (LPS Rats); *In vitro* (LPS/ATP-induced H9c2 cells)	*In vivo*: 200 mg/kg (i.p.)*In vitro*: 400 μg/mL	Wang et al. (2024)

The table summarizes representative natural products categorized by chemical structure, detailing their plant sources, target organs, molecular mechanisms, experimental models, and pharmacological effects. Abbreviations: ALI: acute lung injury; ARDS: acute respiratory distress syndrome; SA-AKI: Sepsis-associated Acute Kidney Injury; SIMI: Sepsis-induced Myocardial Injury; SAHI: Sepsis-associated Hepatic Injury; LPS: lipopolysaccharide; CLP: cecal ligation and puncture; MPO: myeloperoxidase; W/D: Wet/Dry weight ratio; NETs: Neutrophil Extracellular Traps; GSDMD: Gasdermin D; HK2: Hexokinase 2; PFKFB3: 6-phosphofructo-2-kinase/fructose-2, 6-biphosphatase 3; *In vivo*: Animal models (mice/rats); *In vitro*: Cell culture models. References are numbered corresponding to the reference list in the main text.

## 3 Protective effects of natural products on sepsis-associated organ dysfunction

Sepsis-induced multiple organ dysfunction syndrome (MODS) represents the localized manifestation of systemic dysregulation in the immune, metabolic, and coagulation networks. Despite the diverse physiological functions of different organs, they exhibit common cellular and molecular hallmarks under septic pathological conditions: an uncontrolled inflammatory response, a bioenergetic crisis resulting from mitochondrial dysfunction, and aberrant activation of cell death programs. This section will systematically elaborate on how natural products can protect the lungs, kidneys, heart, liver, and gastrointestinal and coagulation systems by precisely targeting these core pathological processes.

### 3.1 Lung injury: macrophage phenotypic plasticity and immunometabolic reprogramming

Sepsis-induced acute lung injury (SILI)/acute respiratory distress syndrome (ARDS) is one of the leading causes of mortality in ICU patients. The core pathophysiological feature lies in the disruption of immune cell homeostasis within the alveolar microenvironment, particularly the phenotypic shift of alveolar macrophages from a homeostasis-maintaining state to a pro-inflammatory state, and the consequent diffuse alveolar damage.

#### 3.1.1 Modulation of macrophage polarization: from inflammation suppression to repair initiation

Macrophages exhibit a high degree of phenotypic plasticity. In the early stage of sepsis, pathogen stimulation drives macrophages to polarize towards the pro-inflammatory M1 phenotype, secreting cytokines such as TNF-α and IL-1β, which disrupt the alveolar-capillary barrier. Tan IIA demonstrates significant immunomodulatory effects in this process. Research by Liu et al. showed that in a rat model induced by cecal ligation and puncture (CLP), Tan IIA significantly downregulated the expression of Bax and Caspase-3 by specifically blocking the ROCK2/NF-κB signaling axis (Liu J. et al., 2022). More critically, it not only inhibited the release of pro-inflammatory factors but also promoted the transformation of macrophages towards the anti-inflammatory/reparative M2 phenotype. Similarly, the triterpenoids taraxasterol (Bu et al., 2022) and hederagenin (Wang and Zhao, 2022) have also been confirmed to block M1 polarization by inhibiting NF-κB nuclear translocation, thereby alleviating pulmonary tissue edema and neutrophil infiltration.

The clinical significance of this finding lies in the fact that while conventional anti-inflammatory therapies (such as corticosteroids) often carry the risk of broad-spectrum immunosuppression, natural products, by promoting the M1-to-M2 macrophage transition, essentially represent an “immune modulation” strategy. This strategy not only mitigates the cytokine storm but also preserves and initiates the repair mechanisms of lung tissue. Consequently, it holds greater value for improving the prognosis of ARDS patients compared to pure immunosuppression.

#### 3.1.2 Targeting immunometabolism: inhibiting glycolytic flux

Immunometabolism research has revealed that inflammatory responses are highly energy-demanding processes, with M1 macrophages heavily relying on aerobic glycolysis (the Warburg effect) to rapidly generate ATP and biosynthetic precursors. Targeting the glycolytic pathway has emerged as a new frontier in the treatment of SILI. The sesquiterpene compound Iso-seco-tanapartholide (IST), isolated from Artemisia argyi, was found to significantly suppress the mRNA and protein expression of PFKFB3, a key rate-limiting enzyme in glycolysis (Kong et al., 2023). This metabolic intervention disrupts the energy supply of M1 macrophages, thereby inhibiting the synthesis of nitric oxide and pro-inflammatory cytokines at the transcriptional level. Furthermore, Cryptotanshinone was shown to alleviate LPS-induced pulmonary edema by activating the cellular energy sensor AMPK, which subsequently inhibits the mTOR pathway-mediated enhancement of glycolysis (Ye et al., 2023). Gastrodin, on the other hand, blocks glycolysis-dependent inflammatory activation by inhibiting Akt phosphorylation and downregulating the expression of another critical enzyme, hexokinase 2 (HK2) (Xie et al., 2023).

This indicates that controlling inflammation through metabolic reprogramming is feasible. Compared to directly blocking downstream cytokines, restricting glycolytic flux represents an “upstream intervention.” By restoring the metabolic homeostasis of immune cells (shifting from glycolysis back to oxidative phosphorylation), it fundamentally limits the capacity for synthesizing inflammatory mediators. This mechanism provides a non-immunosuppressive novel approach for addressing refractory ARDS.

### 3.2 Kidney injury: mitochondrial quality control and enhancement of cellular tolerance

The kidney is one of the organs with the highest resting metabolic rate in the body, and renal tubular epithelial cells are rich in mitochondria to support active reabsorption. Therefore, mitochondrial dysfunction is considered the “Ground Zero” of SA-AKI.

#### 3.2.1 SIRT1-mediated mitochondrial biogenesis and protection

Mitochondria are not only energy factories but also primary targets of oxidative stress. AS-IV demonstrates remarkable mitochondrial protective effects. Studies have confirmed that AS-IV is a potent agonist of the NAD^+^-dependent deacetylase SIRT1. Through SIRT1-mediated deacetylation, AS-IV activates the downstream PGC-1α/Nrf2 axis, promotes mitochondrial biogenesis, and enhances the expression of antioxidant enzymes, thereby significantly improving renal function parameters (serum creatinine, blood urea nitrogen) in septic mice (Sun et al., 2023). In a more complex model involving concomitant hyperhomocysteinemia, AS-IV was further shown to suppress the expression of kidney injury molecules KIM-1 and NGAL by negatively regulating the Gpr97/TPL2 signaling pathway (Xu J. et al., 2024). Similarly, ginsenoside Rg1 maintains the redox balance within renal cells by upregulating SIRT1 and inhibiting NF-κB (Hu et al., 2024).

Traditional treatment for AKI primarily focuses on blood purification to remove toxins, which, however, fails to reverse cellular damage. By activating SIRT1, natural products essentially enhance the “tissue tolerance” of renal cells to septic insult. Maintaining mitochondrial integrity preserves the cellular energy foundation and prevents necrotic cell death triggered by ATP depletion—a pathological reversal that cannot be achieved through supportive therapy alone.

#### 3.2.2 Multi-target network regulation and intervention in cell death

In addition to mitochondrial protection, intervention in programmed cell death is a crucial strategy. Byakangelicin reduces apoptosis in renal tubular epithelial cells by blocking the NF-κB signaling pathway and downregulating pro-apoptotic proteins such as Bax (Hangda et al., 2024). Ginkgolide A inhibits NADPH oxidase-dependent ROS generation by targeting the miR-25/NOX4 axis, thereby blocking oxidative stress-induced cell death (Li et al., 2021). Notably, the mechanism of action of the traditional Chinese medicine Rhizoma Coptidis reflects the characteristics of systems biology. Metabolomics-based studies have found that *Rhizoma Coptidis* extract can simultaneously promote Nrf2 nuclear translocation (antioxidant effect), upregulate PPARα (improving lipid metabolism), and reduce iNOS activity (improving microcirculation). This multi-dimensional regulation is superior to single-target interventions (Zheng et al., 2021).

The pathology of SA-AKI involves a complex interplay of inflammation, ischemia, and metabolic disorders. The efficacy of multi-target drugs like Rhizoma Coptidis suggests that reconstructing the renal metabolic homeostasis network may be more effective than single-target anti-inflammatory or antioxidant interventions. Furthermore, the chemotherapeutic agent paclitaxel, at low doses, has been found to exert anti-inflammatory effects by modulating the lnc-MALAT1/miR-370-3p axis, demonstrating the potential of “drug repurposing” in AKI treatment (Xu et al., 2020). In the context of SA-AKI, resveratrol significantly improves kidney function and possesses robust nephroprotective properties. Notably, resveratrol uniquely restores renal cortical microcirculation and glomerular filtration rate even when administered hours after sepsis onset (Holthoff et al., 2012). Mechanistically, resveratrol activates SIRT1 and SIRT3 deacetylases, which protects mitochondrial function by deacetylating SOD2 and suppresses the NF-κB-driven inflammatory cascade (Xu et al., 2016; Gan et al., 2017). Furthermore, it mitigates renal tubular epithelial cell damage by attenuating endoplasmic reticulum (ER) stress via the IRE1-NF-κB pathway (Zhou et al., 2022) and inhibiting NLRP3 inflammasome activation (Wang et al., 2017). Ultimately, this multi-targeted approach prevents the catastrophic decline of renal function and prolongs survival during septic shock.

### 3.3 Heart and liver: metabolic resuscitation and pyroptosis blockade

The myocardium and liver, serving as the body’s power pump and metabolic hub, are particularly vulnerable to sepsis-induced energy depletion and cellular pyroptosis.

#### 3.3.1 Metabolic resuscitation in sepsis-induced myocardial injury

The essential characteristic of SIMI is myocardial depression, the root cause of which is energy starvation in cardiac cells due to mitochondrial dysfunction. Berberine has been demonstrated to directly target this pathological link. By upregulating the Notch1/Hes1 signaling pathway in myocardial tissue, berberine optimizes mitochondrial structure and significantly increases ATP production, thereby restoring impaired myocardial contractility and improving survival rates in a CLP rat model (Shen et al., 2024). Salvianolic acid B, on the other hand, maintains mitochondrial membrane potential and prevents excessive mitochondrial fission and functional collapse by enhancing the mitochondrial unfolded protein response (mtUPR)—a cellular self-repair mechanism for coping with mitochondrial proteotoxic stress (Chen et al., 2024).

Currently, catecholamine inotropic agents are commonly used in clinical practice for the treatment of SIMI. While these agents can temporarily increase cardiac output, they also significantly elevate myocardial oxygen consumption, potentially exacerbating cellular damage. In contrast, berberine and salvianolic acid B improve cardiac function by enhancing mitochondrial efficiency, representing a “Metabolic Resuscitation” strategy. This approach restores pump function without increasing the oxygen demand burden on the heart, aligning more closely with the pathophysiological requirements for myocardial protection in sepsis.

#### 3.3.2 Hepatic antioxidant defense and pyroptosis regulation

In SAHI, oxidative stress-induced pyroptosis is a primary cause of extensive hepatocyte loss and liver failure. 6-Gingerol enhances the liver’s antioxidant capacity and inhibits macrophage pyroptosis by activating the Nrf2 pathway, thereby mitigating hepatic tissue necrosis (Hong et al., 2020). Prim-O-glucosylcimifugin (Liu L. et al., 2022) and Carvacrol (Xu L. et al., 2024) specifically target the NLRP3 inflammasome. By blocking NLRP3 assembly, they inhibit the activation of Caspase-1 and the cleavage of its downstream effector protein, Gasdermin D (GSDMD), consequently preventing plasma membrane pore formation and the leakage of cellular contents.

Pyroptosis is a lytic form of cell death that releases substantial cellular contents, further amplifying the inflammatory response. The inhibition of the NLRP3/GSDMD axis by natural products essentially disrupts the vicious cycle of “inflammation → cell death → inflammation amplification.” This mechanism is of profound importance for protecting the liver, a vital detoxification organ. Moreover, curcumin significantly reduces the severity of septic-induced liver injury. As comprehensively demonstrated by Zhong et al. in an endotoxemia model, curcumin alleviates sepsis and liver failure by potently suppressing oxidative stress-related inflammation and hepatocyte apoptosis (Zhong et al., 2016). This hepatoprotective mechanism is primarily mediated through the inhibition of the PI3K/AKT and NF-κB signaling pathways, alongside the suppression of the P38/JNK MAPK cascade and modulation of the CYP2E1/Nrf2/ROS axis (Zhong et al., 2016). Consequently, curcumin effectively preserves hepatic architecture, restores antioxidant enzyme activity (such as SOD and GSH), and reduces systemic pro-inflammatory cytokines during inflammatory storms (Zhong et al., 2016).

### 3.4 Gastrointestinal and coagulation: barrier maintenance and immunothrombosis

The disruption of the gastrointestinal barrier and SIC often form a vicious cycle, jointly driving the progression of MODS.

#### 3.4.1 Intestinal barrier integrity and restoration of autophagic flux

The intestine is considered the “motor” of sepsis development. Phellodendrine activates the suppressed autophagic flux by modulating the AMPK/mTOR signaling axis (Ding et al., 2025). This enhanced autophagy not only clears damaged mitochondria and protein aggregates but also protects the tight junctions of intestinal epithelial cells and prevents bacterial translocation by reducing oxidative stress levels. Hesperetin exhibits unique anti-NETs activity. In a ROS-dependent manner, it inhibits NETs formation in the intestine, promoting neutrophil apoptosis instead of the formation of cytotoxic NETs, thereby protecting the intestinal mucosal barrier (Chen et al., 2023).

#### 3.4.2 Precision targeting of immunothrombosis

SIC is not merely a consumptive process of coagulation factors but also involves “immunothrombosis,” where NETs serve as a scaffold for thrombus formation. Forsythiaside B offers a targeted therapeutic approach against this pathological mechanism. In a cecal ligation and puncture rat model, it specifically inhibited peptidylarginine deiminase 4 (PAD4), a key enzyme for histone citrullination and NETs release, thereby improving coagulation parameters and reducing the microcirculatory thrombus burden (He et al., 2022). Parthenolide, by upregulating BRD4/BCL-xL, protected vascular endothelial cells from mitochondria-mediated apoptosis, thereby preserving the anticoagulant properties of the endothelial cell surface (Zhang et al., 2025).

Clinically, conventional heparin anticoagulation therapy carries a significant risk of bleeding and fails to address immune-mediated thrombosis. The natural product strategy targeting PAD4 or NETs offers a novel approach: instead of directly interfering with the coagulation cascade, it improves microcirculation by blocking the “immune-coagulation crosstalk.” Theoretically, this strategy offers greater safety, as it can improve perfusion while avoiding exacerbation of consumptive bleeding due to coagulation factor depletion.

Based on the aforementioned studies, natural products demonstrate a systemic protective pattern in the treatment of SRC. Whether in the lungs, kidneys, heart, or liver, their mechanisms of action are not isolated but converge on several core biological processes: (1) Metabolic reprogramming: restoring energy metabolic homeostasis in immune cells and parenchymal cells via the AMPK/mTOR axis or by inhibiting glycolysis; (2) Mitochondrial quality control: maintaining mitochondrial integrity and enhancing cellular tolerance to septic insult through the SIRT1 and Nrf2 pathways; (3) Interruption of the inflammation-death loop: blocking the positive feedback loop of inflammatory amplification via dual inhibition of the NF-κB and NLRP3/GSDMD axes; (4) Intervention in immunothrombosis: targeting NETs and PAD4 to ameliorate microcirculatory dysfunction. This cross-organ mechanistic consistency further corroborates that the advantage of natural products in treating sepsis lies in their holistic modulation of the systemic pathological network, rather than the amelioration of isolated symptoms.

### 3.5 Brain injury: mitigating sepsis-associated encephalopathy and neuroprotection

The brain is undoubtedly the most critical and vulnerable organ in the human body. During sepsis, systemic inflammation and BBB disruption frequently precipitate SAE, leading to severe cognitive and functional brain disorders. Natural products have demonstrated immense potential in addressing this critical challenge. For example, xanthohumol, a prenylated chalcone, exhibits potent neuroprotective properties and can effectively cross the BBB to modulate microglial activation (Lee et al., 2011; Kirchinger et al., 2019). Mechanistically, xanthohumol exerts profound anti-neuroinflammatory effects by suppressing microglial overactivation and the excessive production of pro-inflammatory cytokines (e.g., IL-1β, IL-6, TNF-α) via the activation of the Nrf2/HO-1 antioxidant defense system (Lee et al., 2011). Furthermore, it effectively inhibits the SIRT1/NF-κB/NLRP3 inflammasome axis, thereby ameliorating cognitive impairment, reducing oxidative stress, and preventing neuronal apoptosis—mechanisms that are highly relevant to SAE pathophysiology (Sun et al., 2021; Lin et al., 2025).

Furthermore, berberine possesses remarkable neuroprotective properties. Recent evidence highlights that berberine effectively alleviates early brain injury and BBB permeability by targeting GSK3β to inhibit CASP1/GSDMD-dependent neuronal pyroptosis (Tan et al., 2026). To further optimize its central nervous system delivery, advanced formulations such as berberine-loaded niosomes have been developed. These nanocarriers significantly reduce brain injury by restoring mitochondrial respiratory chain complexes, regulating mitochondrial dynamics (via MFN-1/2 and DRP-1), and suppressing apoptotic signals (Mansour et al., 2026).

## 4 Common molecular mechanisms across organs: from single-target to network modulation

Through an in-depth analysis of the aforementioned organ-specific studies, it has been observed that despite the distinct physiological functions of the lungs, kidneys, heart, and liver, natural products exhibit significant “Mechanistic Convergence” in improving the functions of these organs. Active compounds with diverse chemical structures often converge on several core signaling hubs. This multi-target regulatory network precisely compensates for the limitations of single-target synthetic drugs (e.g., specific cytokine antagonists) in addressing the systemic pathological network of sepsis. This section will focus on elucidating how natural products restore systemic homeostasis by synergistically modulating three core networks: inflammation, oxidative stress, and immunometabolism.

### 4.1 The inflammatory hub: dual inhibition of NF-κB priming and NLRP3 assembly

NF-κB is a key transcription factor regulating the inflammatory storm in sepsis, responsible for priming the transcription of various pro-inflammatory genes, including TNF-α, IL-6, and Pro-IL-1β. Almost all the natural products reviewed, such as tan IIA (Wang and Zhao, 2022), berberine (Shen et al., 2024), and byakangelicin (Hangda et al., 2024), demonstrate significant inhibitory effects on the TLR4/MyD88/NF-κB signaling axis. However, previous clinical experience indicates that merely inhibiting NF-κB is often insufficient to completely block the inflammatory cascade, as pre-synthesized inflammatory precursors within cells may still be activated through transcription-independent pathways.

This represents the unique advantage of natural products in their pharmacological mechanisms: they often simultaneously intervene in both the post-translational assembly and activation of the NLRP3 inflammasome. For instance, Prim-O-glucosylcimifugin (Liu L. et al., 2022) and Carvacrol (Xu L. et al., 2024) have been demonstrated to specifically block the oligomerization of the NLRP3 protein, thereby inhibiting the cleavage of the effector enzyme Caspase-1 and the maturation and release of IL-1β/IL-18. Furthermore, AS-IV has been found to simultaneously target both NF-κB and NLRP3 (Sun et al., 2023).

This dual inhibition strategy targeting both “priming” and “activation” holds profound clinical significance. The activation of the NLRP3 inflammasome not only leads to the release of cytokines but is also a key driver inducing pyroptosis. As a lytic form of cell death, pyroptosis releases a large number of damage-associated molecular patterns (DAMPs), triggering a secondary inflammatory storm. By simultaneously targeting both NF-κB and NLRP3, natural products essentially block the “inflammation-pyroptosis” positive feedback vicious cycle at the molecular level. This mechanistic redundancy is the key reason for their superior efficacy compared to single NF-κB inhibitors.

### 4.2 Remodeling the cellular defense system: activation of the nrf2/HO-1 pathway

Oxidative stress is a primary driver of mitochondrial dysfunction, lipid peroxidation, and ferroptosis. Rather than simply viewing natural products as “free radical scavengers,” they can be more accurately defined as “upstream inducers” of the endogenous cellular defense system. Nrf2 is the master regulatory transcription factor for cellular defense against oxidative damage. Studies have shown that compounds containing electrophilic groups, such as curcumin (Xu et al., 2019), resveratrol (Zeng et al., 2023), and 6-gingerol (Hong et al., 2020), can modify cysteine residues on the Keap1 protein. This modification promotes the dissociation and nuclear translocation of Nrf2, enabling its binding to the antioxidant response element (ARE) and triggering the expression of downstream antioxidant enzymes (e.g., HO-1, SOD, GSH synthetase).

This point is crucial for understanding the efficacy of natural products. Many small-molecule synthetic antioxidants (such as vitamin C and N-acetylcysteine) have shown limited effectiveness in clinical trials for sepsis, primarily because they neutralize ROS at a 1:1 stoichiometric ratio and become ineffective once depleted. In contrast, natural products activate the Nrf2/HO-1 pathway, thereby initiating the cell’s intrinsic enzymatic defense system. Enzymes possess catalytic properties and are not consumed during the reaction, conferring a significant signal amplification effect and sustained protection. This explains why natural products can effectively maintain organ function even during the late stages of sepsis, when endogenous antioxidants are exhausted.

### 4.3 Emerging frontier: restriction of glycolytic flux and immunometabolic reprogramming

Immunometabolic reprogramming represents the most cutting-edge field in current research on the pathophysiology of sepsis. During the acute phase of sepsis, innate immune cells (such as macrophages and neutrophils) undergo a significant metabolic shift from efficient OXPHOS to inefficient but rapid aerobic glycolysis (the Warburg effect). This switch provides rapid ATP and biosynthetic substrates for cytokine synthesis and phagocytic activity (O'Neill et al., 2016). This metabolic profile constitutes the material basis for sustaining the hyperinflammatory state.

Berberine (Shen et al., 2024), cryptotanshinone (Ye et al., 2023), and IST (Kong et al., 2023) demonstrate remarkable metabolic regulatory capabilities. They force M1 macrophages to shut down excessive glycolytic flux and revert to an OXPHOS state by either activating the energy sensor AMPK or directly inhibiting the key glycolytic enzyme PFKFB3. Additionally, gastrodin restricts glucose uptake by inhibiting HK2 (Xie et al., 2023).

This mechanism targets the energetic foundation of sepsis pathology. If anti-inflammatory drugs are considered to block the inflammatory “signal,” then metabolic modulators essentially cut off the “substrate supply” for inflammation. By restricting glycolytic flux, the natural product not only suppresses lactate accumulation (where lactate itself acts as a pro-inflammatory signal) but also promotes the polarization of macrophages toward the M2 phenotype, which relies on OXPHOS. This “metabolic reprogramming” strategy effectively quells excessive inflammatory responses without compromising the body’s fundamental immune defenses, thereby providing the necessary metabolic environment for tissue regeneration during the recovery phase of sepsis.

### 4.4 Interaction and network integration of mechanisms: from independent pathways to systemic homeostasis

**FIGURE 2:**
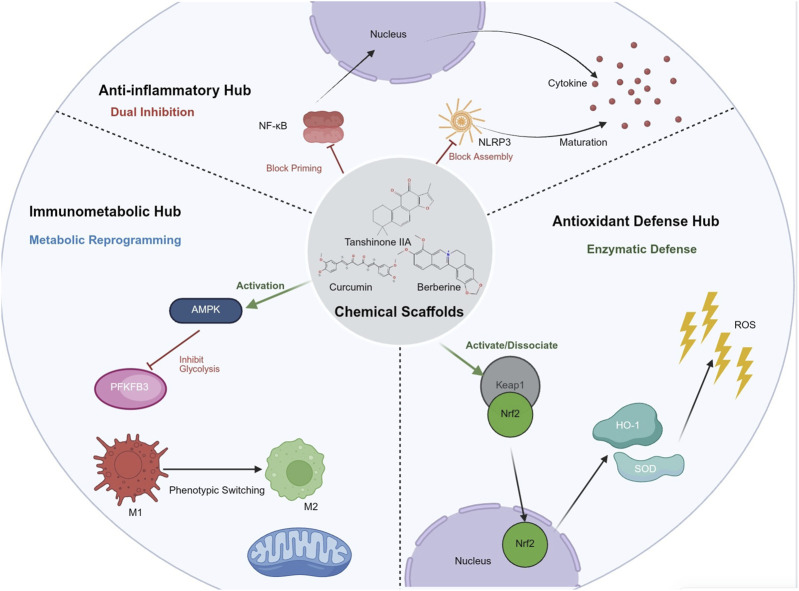
Integrated Molecular Mechanisms of Natural Products in Treating Sepsis: Synergistic Modulation of Inflammation, Oxidation, and Immunometabolism. (Created with BioRender.com.). Note: Unlike single-target synthetic drugs, natural products exert therapeutic effects through polypharmacological networks targeting three convergent signaling hubs: (1) Anti-inflammatory Hub: Dual inhibition of NF-κB transcriptional priming and NLRP3 inflammasome post-translational assembly, blocking the release of TNF-α, IL-6, and IL-1β. (2) Antioxidant Defense Hub: Activation of the Nrf2/HO-1 axis, which acts as an upstream inducer of the cellular enzymatic defense system to scavenge ROS and protect mitochondrial integrity. (3) Immunometabolic Reprogramming: Regulation of the AMPK/mTOR axis and inhibition of glycolytic enzymes (e.g., PFKFB3), shifting immune cells from a pro-inflammatory glycolytic phenotype to a restorative oxidative phosphorylation phenotype. Additionally, the figure illustrates organ-specific protection, such as the inhibition of NETs in coagulation disorders and the regulation of macrophage polarization (M1-to-M2 transition). This multi-target strategy restores systemic homeostasis by simultaneously intervening in crosstalk between inflammation, oxidative stress, and metabolic dysregulation.

It must be emphasized that the aforementioned three core mechanisms—inflammatory regulation, antioxidant defense, and immunometabolism—do not operate in isolation during the pathophysiological process. Instead, they are intricately intertwined through complex signaling crosstalk, forming a regulatory network in which a single perturbation can affect the entire system (see Figure 2). The therapeutic advantage of natural products lies precisely in their ability to simultaneously target these critical crosstalk nodes, thereby disrupting the vicious cycle: (1) Decoupling the oxidative-inflammatory axis: Mitochondrial ROS serves as a key upstream trigger for NLRP3 inflammasome activation (Zhou et al., 2011). By activating the Nrf2/HO-1 pathway to scavenge ROS, natural products not only protect organelles but also block the assembly signal for NLRP3 at its source. (2) Blocking the metabolic-inflammatory axis: Intermediate metabolites generated by glycolysis (e.g., succinate) can stabilize HIF-1α, thereby promoting IL-1β transcription (Tannahill et al., 2013). Natural products inhibit glycolytic flux via AMPK, essentially severing this “metabolic-inflammatory” positive feedback loop. (3) Antagonistic interaction of transcription factors: The NF-κB p65 subunit can competitively bind to Keap1 or directly inhibit the transcriptional activity of Nrf2 (Liu et al., 2008). By inhibiting NF-κB nuclear translocation, natural products relieve its suppression of Nrf2, thereby restoring the impaired cellular antioxidant capacity.

In summary, the molecular basis of natural products in the treatment of sepsis extends far beyond simple “multi-target superposition.” It lies in their synergistic modulation of the “NF-κB/NLRP3 inflammatory axis,” the “Nrf2/HO-1 antioxidant axis,” and the “AMPK/mTOR immunometabolic axis.” This regulatory model, grounded in “network pharmacology,” not only explains why a single natural product can exert broad, multi-organ protective effects but also untangles its intrinsic advantage over single-target synthetic drugs (which often fail due to compensatory mechanisms). Specifically, natural products do not merely attempt to “block” a specific physiological process; instead, they are dedicated to “remodeling” the systemic homeostasis of the organism under the assault of infection.

## 5 Limitations and translational challenges: critical barriers to clinical translation

Although natural products have demonstrated significant protective effects against SRCs in preclinical models, the vast majority of research remains confined to cellular or rodent experiments. Translating these experimental findings into clinically applicable therapeutic agents necessitates overcoming critical barriers, including poor pharmacokinetic properties, insufficient safety evaluations, and heterogeneity in clinical evidence.

### 5.1 The druggability bottleneck: bioavailability and pharmacokinetic profiles

The primary translational challenge for natural products, particularly polyphenols and flavonoids, lies in their poor druggability. Many compounds exhibit nanomolar (nM) activity in *ex vivo* assays but fail to achieve effective plasma concentrations *in vivo*.

Most bioactive natural products, such as curcumin, resveratrol, and luteolin, possess high hydrophobicity, resulting in extremely low oral bioavailability. More critically, these compounds are rich in phenolic hydroxyl groups, making them substrates for Phase II metabolic enzymes in the liver and intestine, such as UDP-glucuronosyltransferases (UGTs) and sulfotransferases (SULTs). Studies have demonstrated that curcumin undergoes extensive glucuronidation rapidly after oral administration, leading to extremely low plasma concentrations of the parent drug (often below 1%) (Anand et al., 2007).

The substantial disparity between *in vivo* and *ex vivo* concentrations suggests that mechanistic conclusions derived solely from *ex vivo* cellular experiments, which typically involve direct exposure to high concentrations of the parent drug, may not be directly extrapolated to the *in vivo* context. Future research must prioritize the evaluation of metabolite activity or focus on developing nanodelivery systems (e.g., liposomes, polymeric micelles) and prodrug strategies to enhance drug solubility and metabolic stability, thereby ensuring that effective therapeutic concentrations are achieved in target organs (Mcclements, 2020; Luo et al., 2014).

### 5.2 Safety considerations: therapeutic window and organ toxicity

“Natural origin” does not equate to “clinical safety.” Some natural products possess a narrow therapeutic window, and their dose-toxicity relationship is non-linear.

In the pathological state of sepsis, patients often present with acute liver and kidney dysfunction (SA-AKI/SAHI). This leads to a significant decrease in drug clearance and a prolonged plasma half-life, thereby drastically increasing the risk of drug accumulation and toxicity. For instance, while emodin is effective against lung injury, high doses of emodin carry potential hepatotoxicity and nephotoxicity, which may induce hepatic sinusoidal obstruction syndrome (Dong et al., 2016). Similarly, although triptolide exhibits potent anti-inflammatory effects, its severe reproductive toxicity and bone marrow suppression limit its clinical application (Xi et al., 2017). Furthermore, natural products often act as inducers or inhibitors of cytochrome P450 enzymes (CYP450) and may interact with commonly used clinical antibiotics and anticoagulants, leading to serious adverse drug-drug interactions (Fasinu et al., 2012).

This necessitates the establishment of rigorous pharmacokinetic/pharmacodynamic models during clinical translation, with a particular emphasis on reassessing drug safety in the context of impaired organ function. Future research should clearly define the “no-observed-adverse-effect level” for each compound and explore strategies to reduce inherent toxicity through structural modifications.

### 5.3 Heterogeneity of clinical evidence and the need for precision medicine

Current clinical evidence supporting the use of natural products for sepsis treatment predominantly originates from single-center, exploratory trials with small sample sizes. These studies often employ complex compound preparations (e.g., Xuebijing), making it difficult to definitively attribute therapeutic efficacy to any specific monomer.

Existing mechanistic studies heavily rely on murine models (e.g., CLP or LPS-induced). However, mice exhibit approximately a 10,000-fold greater tolerance to endotoxin compared to humans, and their genomic response patterns differ profoundly from those observed in human sepsis (Seok et al., 2013). More importantly, sepsis is not a single disease entity but rather a highly heterogeneous clinical syndrome. Different patients may present with distinct immunological endotypes, such as a hyper-inflammatory state or a state of immune paralysis (Seymour et al., 2019). Attempting to treat all sepsis patients with a single natural product (e.g., anti-inflammatory flavonoids) is inconsistent with pathophysiological logic, which likely constitutes a primary reason for the failure of previous clinical trials.

Future clinical trial designs must incorporate the principles of precision medicine. Patients should be stratified using biomarkers (e.g., ferroptosis markers, inflammatory cytokine profiles) to identify subpopulations most likely to benefit from specific mechanism-based therapies (e.g., Nrf2 agonists for the high-oxidative-stress subtype) (Stanski and Wong, 2020). Only through such an “enrichment strategy” can the clinical efficacy of natural products be objectively validated.

In summary, the clinical translation of natural products is constrained by low bioavailability, potential organ toxicity, and heterogeneous clinical evidence. Overcoming these barriers requires multidisciplinary collaboration: leveraging medicinal chemistry to optimize drug-like properties, utilizing toxicology to define safety margins, and applying systems biology to guide the design of precision clinical trials.

## 6 Conclusion and future perspectives

### 6.1 Summary: from chemical diversity to systemic regulation

This review systematically elucidates the scientific rationale for natural products as potential therapeutic agents for SRCs. Analysis based on chemical structures indicates that polyphenols, terpenoids, alkaloids, and quinones, by virtue of their unique physicochemical properties, can precisely target key nodes within the pathological network of sepsis. Their core mechanisms extend beyond a singular anti-inflammatory effect, achieving systemic protection of critical organs such as the lungs, kidneys, heart, and liver through the synergistic modulation of the NF-κB/NLRP3 inflammatory axis, the Nrf2/HO-1 antioxidant system, and the AMPK/mTOR immunometabolic network. This “multi-target, multi-pathway” pharmacological profile endows them with unparalleled advantages over synthetic single-target drugs in confronting the complexity of sepsis, a disease involving the collapse of systemic homeostasis.

### 6.2 Discussion and future perspectives translating SAR insights into rational drug design

To bridge the translational gap between preclinical promise and clinical efficacy in sepsis management, future research paradigms must decisively pivot from the empirical screening of crude extracts toward SAR-guided lead compound optimization and targeted delivery. As elucidated in our comparative analyses, the innate limitations of natural products—such as poor aqueous solubility, rapid first-pass metabolism, and narrow therapeutic windows—can be systematically circumvented through rational structural modifications. The strategic blueprints are already evident: the targeted methylation of flavonoid hydroxyl groups to overcome the “bioavailability paradox,” the manipulation of terpenoid spatial conformations (e.g., planar *versus* puckered rings) to fine-tune kinase binding affinities, and the exploitation of quaternary ammonium charge states to direct intracellular mitochondrial tropism.

Moving forward, realizing the full clinical potential of these optimized scaffolds requires integrating traditional medicinal chemistry with modern systems biology and advanced nanomedicine. For instance, network pharmacology and metabolomics can be leveraged to map the multi-target synergistic networks of these natural derivatives against the highly heterogeneous pathophysiology of sepsis. Furthermore, as bacterial drug resistance increasingly compromises conventional antibiotic therapies in critical care settings, natural products that are structurally optimized for dual immunomodulatory and anti-microbial effects represent a highly promising frontier. When coupled with stimuli-responsive or biomimetic nanocarriers—which enable sustained release and targeted accumulation at inflammatory sites—these compounds can safely navigate the biphasic immune responses of sepsis. Ultimately, by combining artificial intelligence-driven structural design with rigorous *in vivo* pharmacokinetic validations, researchers can engineer semi-synthetic analogs that preserve the holistic, multi-pathway advantages of natural scaffolds while delivering precise, pharmacokinetically stable interventions to halt sepsis-associated organ dysfunction.

### 6.3 Roadmap: crossing the translational gap

To translate the experimental potential of natural products into clinical reality, future research should focus on the following three strategic directions: (1) Innovation in Drug Delivery Systems: To address the poor water solubility and rapid metabolism of compounds such as polyphenols, there should be a strong emphasis on developing targeted nano-delivery systems (e.g., mannose-modified liposomes targeting pulmonary macrophages, or chitosan nanoparticles targeting renal tubules). This approach can not only increase local drug concentrations but also reduce the risk of toxicity associated with systemic exposure. (2) Biomarker-Based Precision Clinical Trials: Moving away from the “one-size-fits-all” clinical trial model (Perner et al., 2017). Multi-omics technologies (transcriptomics, metabolomics) should be utilized to identify specific sepsis subtypes (e.g., immunosuppressive vs. cytokine storm types) and to design biomarker-driven RCTs accordingly, enabling personalized and precise treatment with natural products. (3) Exploration of Combination Therapy Strategies: Given the threat of bacterial drug resistance, exploring the synergistic effects of natural products with antibiotics (e.g., by disrupting bacterial biofilms or inhibiting efflux pumps) is of significant strategic importance. Furthermore, investigating the combination of natural products with modern life support technologies (e.g., Continuous Renal Replacement Therapy, CRRT) to explore their anti-inflammatory and organ-protective effects during *ex vivo* circulation represents a highly valuable clinical direction.

### 6.4 Final remarks

Natural products are no longer merely a continuation of traditional empirical knowledge; they have become a profound source of lead compounds in the modern drug library for critical care medicine. With the increasing integration of structural biology, network pharmacology, and nanomedicine, it is reasonable to believe that scientifically optimized and rigorously validated natural product monomers will ultimately bridge the gap from the laboratory to the bedside, emerging as important therapeutic agents for improving the prognosis of sepsis patients worldwide.
